# Disseminated peritoneal leiomyomatosis with chronic constipation: a case report

**DOI:** 10.1186/1752-1947-8-114

**Published:** 2014-04-03

**Authors:** Savas Bayrak, Esra Pasaoglu, Ekrem Cakar, Hasan Bektas, Sukru Colak, Mert Mahsuni Sevinc, Erdem Kinaci

**Affiliations:** 1 Department of General Surgery, Istanbul Training and Research Hospital, Kasap İlyas Mah. Org. Abdurrahman Nafiz Gürman Cd. PK: 34098., Istanbul (212) 4596000, Turkey; 2 Department of Pathology, Istanbul Training and Research Hospital, Kasap İlyas Mah. Org. Abdurrahman Nafiz Gürman Cd. PK: 34098., Istanbul (212) 4596000, Turkey

**Keywords:** Abdominal distention, Abdominal pain, Chronic constipation, Disseminated peritoneal leiomyomatosis

## Abstract

**Introduction:**

Disseminated peritoneal leiomyomatosis is a rare disease. Almost all disseminated peritoneal leiomyomatosis cases described in the literature are associated with a gynecological disorder or a mass in the abdominal cavity. Disseminated peritoneal leiomyomatosis with only chronic constipation has not been reported in the English literature. We present a case of a patient with disseminated peritoneal leiomyomatosis who manifested solely with chronic constipation.

**Case presentation:**

A 49-year-old premenopausal nulliparous Caucasian woman was admitted with complaints of abdominal distention and chronic constipation. Open subtotal colectomy with ileorectal anastomosis was performed. There were diffuse nodular and polypoid tumor formations in her colonic mesoderm. Based on morphological and pathological evaluation of the resection material, she was diagnosed with disseminated peritoneal leiomyomatosis.

**Conclusions:**

In general, disseminated peritoneal leiomyomatosis is seen in women who are of childbearing age with estrogen hypersecretion. Preoperative diagnosis of disseminated peritoneal leiomyomatosis is almost impossible and it can be confused with disseminated intra-abdominal malignancies. There are no specific methods to diagnose disseminated peritoneal leiomyomatosis in a preoperative period.

## Introduction

Disseminated peritoneal leiomyomatosis (DPL) is a rare disease which was first defined in 1952 by Willson and Peale [1-3]. It is generally seen in women who are of childbearing age with estrogen hypersecretion [1]. Preoperative diagnosis of DPL is almost impossible; DPL can be confused with disseminated intra-abdominal malignancies [4]. The histogenesis of DPL is not clear due to the rarity of the disease, although is thought to be associated with benign smooth muscle proliferation originating from the multicentric metaplasia of the peritoneal surfaces [5,6]. In this study, we report a case of a patient with DPL whose main complaint was chronic constipation.

## Case presentation

A 49-year-old premenopausal nulliparous Caucasian woman was admitted with the complaints of abdominal distention and constipation. She indicated that she had been defecating once every 7 to 10 days, frequently with the help of a laxative, and had had serious abdominal pain intermittently since childhood. She had undergone an exploratory laparotomy for ileus 1 year ago. The related surgery report stated that her colon had been extensively dilated and elongated, and there had been massive fecal impactions obstructing her colon. The impactions had been disintegrated and removed, and no other pathology had been observed in the surgery.

Some laboratory and screening tests were carried out for differential diagnosis of the patient. Laboratory test results were nonspecific and tumor markers were within normal ranges. Anal manometry confirmed the presence of basal waves and rectosphincteric reflex. A barium enema showed dilation of her entire colon. Her colonic transit time was found to be prolonged. Her mucosal structure had a normal appearance at colonoscopy and there were no extra pathological findings in defecography. Informed consent was obtained and she underwent a subtotal colectomy including ileorectal anastomosis due to chronic constipation. Significant elongation and dilation of her colon were observed in the surgery. There were diffuse nodular and polypoid tumor formations in the colonic mesoderm. Since no early and late surgical complications occurred, she was discharged from our hospital at postoperative day 5 with surgical recovery.

**Figure 1:**
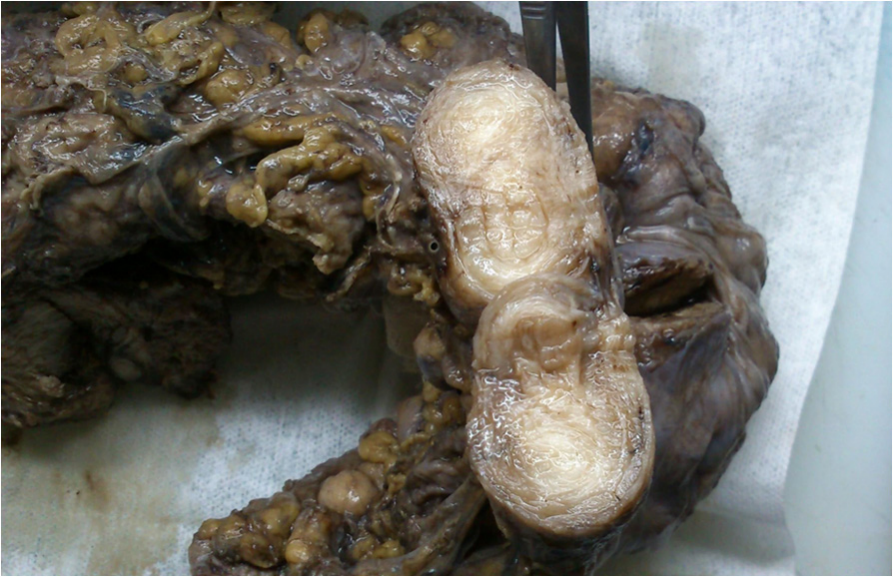
Nodular lesion, grey-white in color localized in the mesenteric adipose tissue.

**Figure 2:**
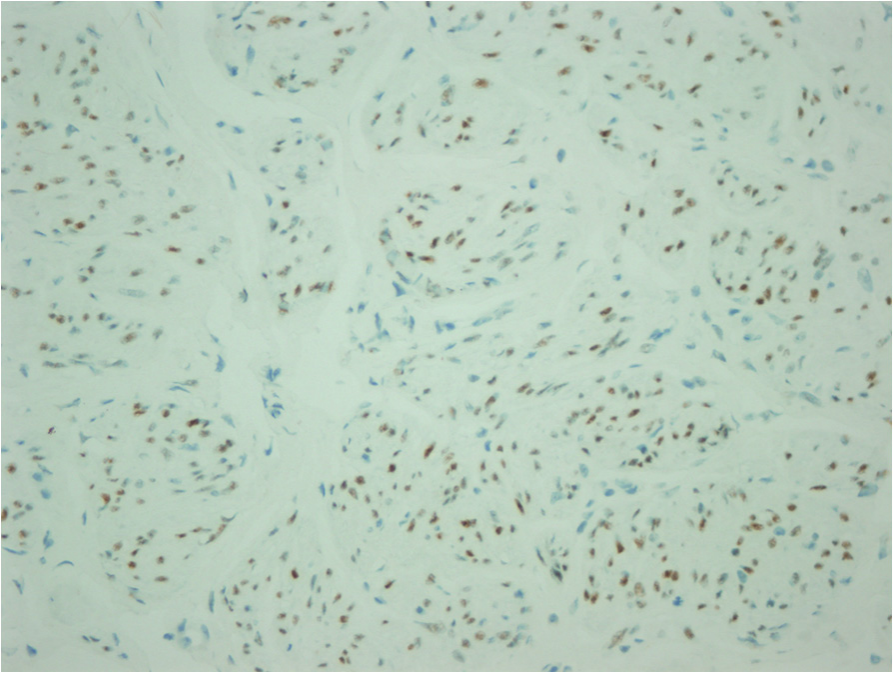
Disorganized smooth muscle bundles in the muscularis propria of the colon wall.

**Figure 3:**
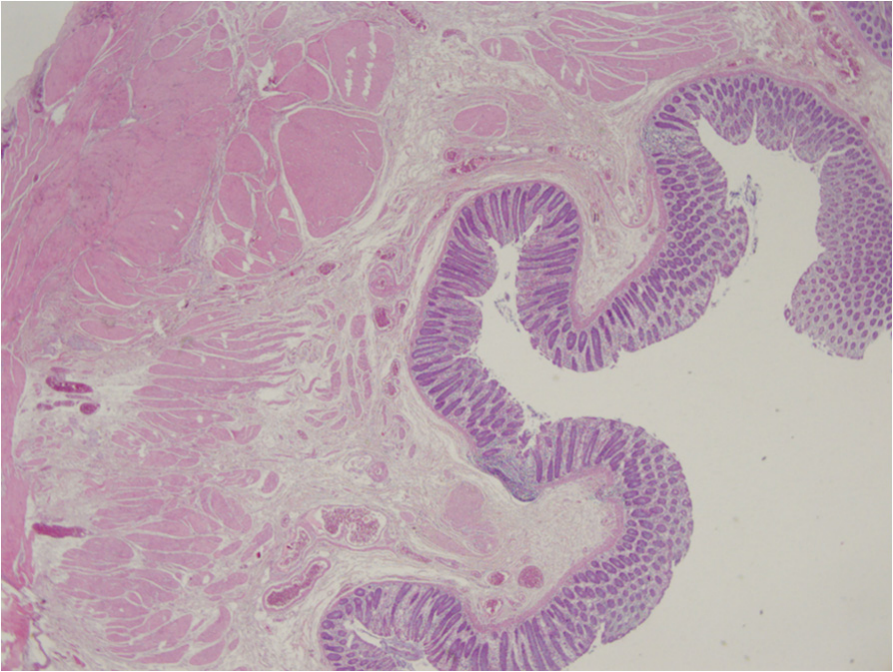
Lesion localized in the mesenteric surface with the surrounding tissue.

**Figure 4:**
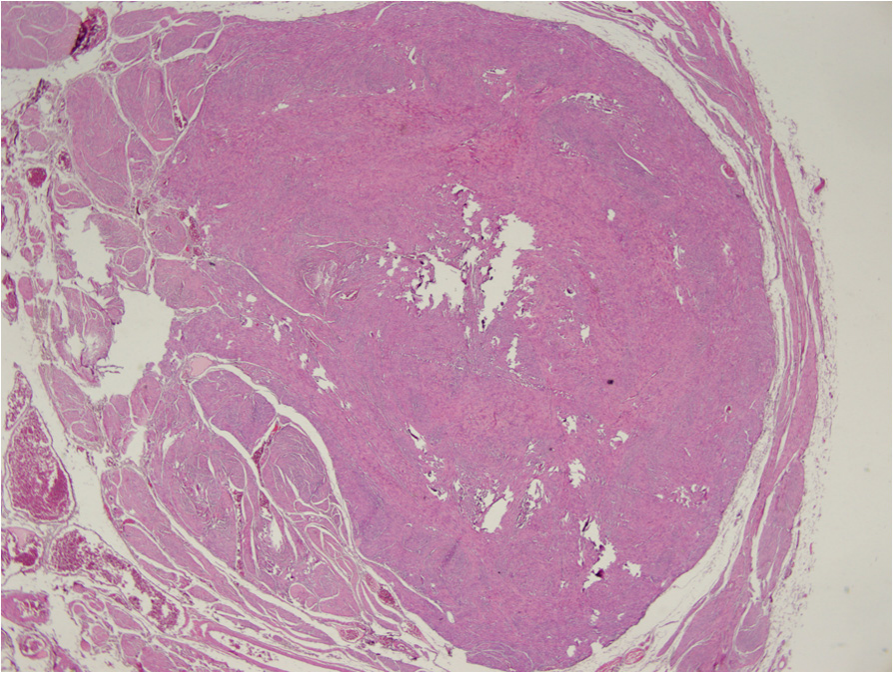
Immunohistochemical staining showing desmin positivity.

**Figure 5:**
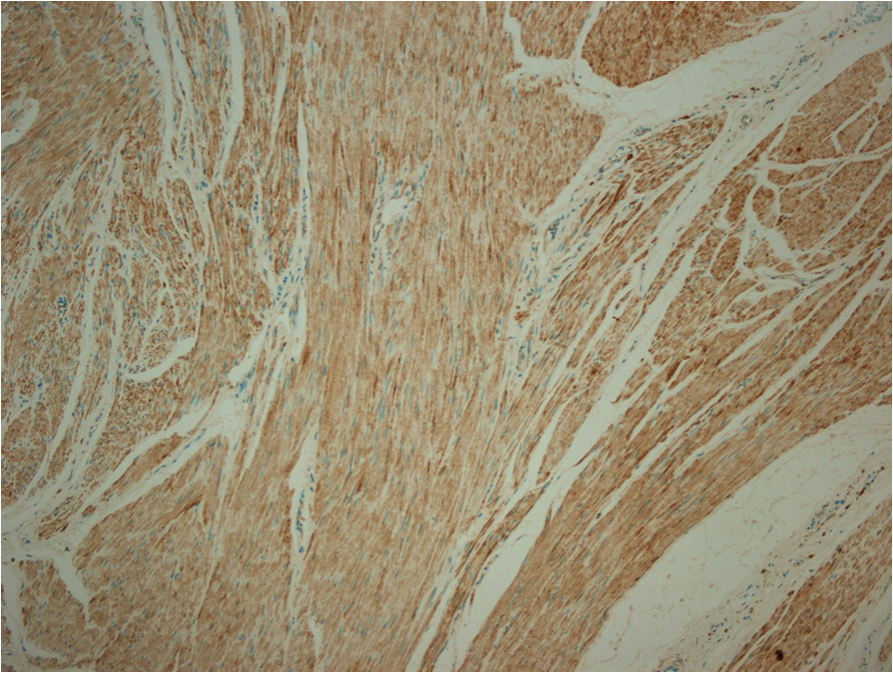
Estrogen receptor positivity.

The resection material was composed of distal ileum, cecum, ascending colon, transverse colon and sigmoid colon. Multiple nodular formations with a hard elastic consistency were observed in the mesenteric adipose tissue (Figure 1). They had a maximum size of 10 × 4 × 2.5cm; they were spherical-ovoid or irregular in shape, combined with each other and attached to her bowel wall by thin fibrous bands. The cut surface had a fibrous structure and appeared gray-white in color. Colonic mucosa was normal, however, her bowel walls were hardened and partially thickened (about 1.5cm). Her parietal and pelvic peritoneum, mesoderm of the small intestine, uterus and ovaries were macroscopically normal. There were disorganized, irregular, partly dissociated smooth muscle bundles in the muscularis propria (Figure 2). Lesions in the mesenteric adipose tissue were composed of fusiform cells (with eosinophilic cytoplasm and ovoid-fusiform nucleus) that formed bundles crossing each other (Figure 3). The margins of the lesions were irregular. Mitosis, nuclear atypia, necrosis and Ki-67 ratio were evaluated to differentiate her case from a leiomyosarcoma (LMS). Mitosis, nuclear atypia and necrosis were not observed and her Ki-67 ratio was 1%. To differentiate her case from a gastrointestinal stromal tumor (GIST), we evaluated actin, desmin, S100, CD117 and CD34. Actin and desmin were positive (Figure 4), whereas S100, CD117 and CD34 were negative in our patient. We also evaluated estrogen and progesterone receptors in order to support DPL diagnosis. Both receptors were positive (Figure 5). The morphological appearance and the results of immunohistochemical studies were compatible with DPL.

## Discussion

We report the case of a patient affected with the rare disease DPL. It is difficult to diagnose DPL due to its resemblance to malignant conditions. However, there are some differences to consider such as in the cases of LMS and GIST. LMS has a higher mitotic index and shows nuclear atypia, tumor necrosis and infiltrative patterns [6]. Our case did not show mitosis, nuclear atypia and necrosis on microscopic evaluation. The Ki-67 ratio was also 1% in our case. GISTs do not commonly have smooth muscle cells in the nodules and show immunohistochemical expression of CD117 and CD34 [1,7]. CD117 and CD34 were negative in our patient microscopically. By contrast, on microscopic evaluation actin, desmin, estrogen and progesterone receptors are expected to be positive in patients with DPL [1]. Actin, desmin, estrogen and progesterone receptors were positive in our patient. Thus, the findings were compatible with DPL.

Female patients with DPL in cases reported in the literature were generally pregnant, using oral contraceptives or had malignant ovarian tumors expressing high estrogen and/or progesterone at the time of diagnosis [6,8]. Moreover, estrogen and progesterone receptors were immunohistochemically positive in the majority of patients with DPL [8-11]. The estrogen receptor was also positive in our case. However, the disease can occur in males and postmenopausal females [11-13].

Some cases of DPL showed malignant transformation [14]. There can be a relation between malignant transformation and p53 overexpression. Yamaguchi *et al.* found that p53 was strongly positive in transformed malignant cells [14]. Since patients with DPL with malignant change have been reported to have poor prognosis, the expression of p53 can also be checked in patients with DPL [14]. Surgery is the primary treatment of DPL, but there is a little information about the progressive tumors of the peritoneum and metastatic tumors of DPL. Systemic chemotherapy was also considered a treatment option for patients with unresectable or metastatic disease [15]. Almost all DPL cases described in the literature are associated with a gynecological disorder or a mass in the abdominal cavity. However, our patient did not show these clinical features. Chronic constipation was the only clinical complaint in the patient. The coexistence of chronic constipation and DPL could be coincidental. However, chronic constipation could be caused by DPL. Since the mesenchymal and the large intestinal system were involved, it is possible that the whole intestinal system could not work normally. DPL with only chronic constipation has not been reported before. Therefore, our patient presents an unusual case of the disease and should be followed for DPL.

## Conclusions

It is important to diagnose DPL because some cases of DPL can show malignant transformation. Pathological evaluation is critical for diagnosing the disease. Other signs and symptoms, such as chronic constipation, may mask the diagnosis of DPL.

## Consent

Written informed consent was obtained from the patient for publication of this case report and accompanying images. A copy of the written consent is available for review by the Editor-in-Chief of this journal.

## Abbreviations

DPL: Disseminated peritoneal leiomyomatosis; GIST: Gastrointestinal stromal tumor; LMS: Leiomyosarcoma.

## Competing interests

The authors declare that they have no competing interests.

## Authors’ contributions

SB, EC and EP were involved in the conception of the report, review of literature, manuscript preparation and submission. HB and SB performed the surgery. SC, MMS and EK were responsible for manuscript critique and review. All authors read and approved the final manuscript.
